# Enhanced Water Splitting by Fe_**2**_O_**3**_-TiO_**2**_-FTO Photoanode with Modified Energy Band Structure

**DOI:** 10.1155/2013/723201

**Published:** 2013-12-31

**Authors:** Eul Noh, Kyung-Jong Noh, Kang-Seop Yun, Bo-Ra Kim, Hee-June Jeong, Hyo-Jin Oh, Sang-Chul Jung, Woo-Seung Kang, Sun-Jae Kim

**Affiliations:** ^1^Institute/Faculty of Nanotechnology and Advanced Materials Engineering, Sejong University, Seoul 143-747, Republic of Korea; ^2^Department of Environmental Engineering, Sunchon National University, Suncheon, Jeonnam 540-742, Republic of Korea; ^3^Department of Metallurgical & Materials Engineering, Inha Technical College, Incheon 402-752, Republic of Korea

## Abstract

The effect of TiO_2_ layer applied to the conventional Fe_2_O_3_/FTO photoanode to improve the photoelectrochemical performance was assessed from the viewpoint of the microstructure and energy band structure. Regardless of the location of the TiO_2_ layer in the photoanodes, that is, Fe_2_O_3_/TiO_2_/FTO or TiO_2_/Fe_2_O_3_/FTO, high performance was obtained when **α**-Fe_2_O_3_ and H-TiNT/anatase-TiO_2_ phases existed in the constituent Fe_2_O_3_ and TiO_2_ layers after optimized heat treatments. The presence of the Fe_2_O_3_ nanoparticles with high uniformity in the each layer of the Fe_2_O_3_/TiO_2_/FTO photoanode achieved by a simple dipping process seemed to positively affect the performance improvement by modifying the energy band structure to a more favorable one for efficient electrons transfer. Our current study suggests that the application of the TiO_2_ interlayer, together with **α**-Fe_2_O_3_ nanoparticles present in the each constituent layers, could significantly contribute to the performance improvement of the conventional Fe_2_O_3_ photoanode.

## 1. Introduction

Green energy sources have been extensively investigated to replace the fossil fuels due to their inherent problems of pollution and limited resources [1]. Among them, hydrogen (H_2_) gas was one of the most actively studied energy sources owing to its abundance, high specific energy capacity, and environmentally friendliness [2–4]. Hydrogen can be produced by using hydrocarbons such as fossil fuels, natural gas, and water. Production of hydrogen gas by electrolysis of water has been known to be the most efficient way [5–7]. Energy required to generate hydrogen and oxygen by electrolysis of water can be supplied through sun light. For the sun light to be effectively utilized, electrodes having functions of photoabsorbent and catalyst need to be employed for electrolysis of water. Photoelectrochemical (PEC) system is an efficient approach to produce hydrogen gas from water by utilizing an unlimited resource of the sun light without generating environmentally deleterious byproducts. With the development of PEC system, much attention has been paid to the fabrication of high efficient photoelectrode for water splitting [4, 8–10]. Among other things, materials extensively studied for the photoelectrode were Co [11, 12], Co-Pi [13, 14], IrO_2_ [15], TiO_2_ [16–18], CuO [19], WO_3_ [20], Fe_2_O_3_ [21], and so forth.

In particular, more interest has been drawn to Fe_2_O_3_ material which could harvest visible part of solar spectrum [21–23]. However, Fe_2_O_3_ has some critical issues to be resolved for the application to the PEC system as photoelectrode such as electron-hole recombination. Several approaches have been taken to reduce the recombination; application of nanostructured materials, doping with appropriate materials, and so forth. Photocurrent density generated with the Fe_2_O_3_ nanorods and nanowires was reported to have 1.3 mA/cm^2^ [21] and 0.54 mA/cm^2^ at 1.23 (*V* versus RHE) [22], respectively. On the other hand, Fe_2_O_3_ photoanode doped with Ti and Si showed a little better performance of 1.83 mA/cm^2^ [24] and 2.2 mA/cm^2^ at 1.23 (*V* versus RHE) [25], respectively. However, the photocurrent density of Fe_2_O_3_ photoanode modified with the nanostructures and doping was found to be still far below the theoretical value of 12.6 mA/cm^2^ at 1.23 (*V* versus RHE). From our previous work, we reported a high photocurrent density of 1.32 mA/cm^2^ at 1.23 (*V* versus RHE) with Fe_2_O_3_/FTO photoanode without any doping [26], synthesized by a simple process of dip coating and short-time heat treatment at 500°C of nanosized Fe_2_O_3_ on the FTO substrate. Our results confirmed the importance of microstructure of Fe_2_O_3_ to the reduction of electron-hole recombination, which could be modified and optimized by the coating amount of Fe_2_O_3_ and following heat treatment conditions [27]. Taking advantage of photocatalytic effect of TiO_2_, Fe_2_O_3_/TiO_2_/FTO photoanode was also fabricated in another study. From the energy band structure viewpoint of the photoanode, the electrons generated on the Fe_2_O_3_ film should overcome a barrier to be transferred to FTO, probably deteriorating the performance [28]. However, the photoanode showed the opposite result of much higher photocurrent density of 4.81 mA/cm^2^ at 1.23 (*V* versus RHE) [29].

In this current work, the effect of microstructure and energy band structure of the photoanodes with the different arrangement of the constituent elements (e.g., TiO_2_/Fe_2_O_3_/FTO, Fe_2_O_3_/TiO_2_/FTO) on the performance was investigated and discussed in relation with the electrons transfer in the photoanode.

## 2. Experimental Details

FTO glasses (Asahi Glass Co.) as a conducting substrate of Fe_2_O_3_ photoanode film for water splitting was at first etched for 20 min using Piranha solution (7 : 3 = 70% conc. H_2_SO_4_ : 30% H_2_O_2_) to make them have fresh surface and then were dipped simply to make H-TiNT (hydrogen titanate nanotube) particles supported in aqueous Fe(NO_3_)_3_ solution (corresponding to Fe_2_O_3_ precursor) or H-TiNT particles dispersed solution (corresponding to TiO_2_ precursor particles). In this study, various photoanode arrangements such as Fe(NO_3_)_3_/FTO, Fe(NO_3_)_3_/H-TiNT/FTO, and H-TiNT/Fe(NO_3_)_3_/FTO were prepared. Coated Fe(NO_3_)_3_ and H-TiNT particles were transformed into Fe_2_O_3_ and TiO_2_ phases, respectively, with heat treatments at 500°C for 10 min in air. In other words, for the performance improvement of Fe_2_O_3_ film, the arrangements with H-TiNT interlayer incorporated in between Fe(NO_3_)_3_ and FTO and with H-TiNT top layer on the Fe(NO_3_)_3_/FTO were tried. All aqueous solutions in this experiment were prepared using distilled water with 1.8 MΩ.

To make H-TiNT interlayer (finally Fe_2_O_3_/TiO_2_/FTO arrangement), the FTO glass after having been surface-treated for 20 min in 0.2 M polyethyleneimine (PEI, Aldrich Co.) aqueous solution containing positively charged ions was used as a transparent conductive substrate. First, the surface-pretreated FTO glass was immersed for 20 min in an aqueous 10 g/L H-TiNT particle solution dispersed together with 0.2 M tetrabutylammonium hydroxide (TBAOH, Aldrich Co.) to produce negatively charged ions. Afterwards, using the same method, an H-TiNT-treated film was subsequently immersed in 0.2 M polydiallyldimethylammonium chloride (PDDA, Aldrich Co.) aqueous solution, which contained positively charged ions. The obtained H-TiNT/FTO glass was dried under UV-Vis light irradiation (Hg-Xe 200 W lamp, Super-cure, SAN-EI Electric) to remove water and all surfactants, such as PEI, TBAOH, and PDDA using photocatalytic removal reaction occurred by H-TiNT particles with optical energy bandgap of 3.5 eV [24], without any sintering. Then, for the Fe(NO_3_)_3_ nanoparticle coating process, the dried H-TiNT/FTO substrates were dipped in an aqueous 1.0 M Fe(NO_3_)_3_ solution with dipping times of 12 hrs. For formations of H-TiNT top layer on Fe(NO_3_)_3_/FTO films (finally TiO_2_/Fe_2_O_3_/FTO arrangement), the precursor solution of Fe_2_O_3_ film supported was made of 1.0 M Fe(NO_3_)_3_·9H_2_O and 0.2 M TBAOH (tetrabutylammonium hydroxide, Aldrich) for dipping fresh FTO substrate for 12 hrs. After that, obtained Fe(NO_3_)_3_/FTO were dried at 80°C for 12 hrs. For formation of H-TiNT/Fe(NO_3_)_3_/FTO films, repetitive self-assembling of oppositely charged ions in an aqueous solution was applied to coat directly the H-TiNT particles using the same process explained above. All dipping process was carried out at room temperature in air.

All heat treatment was done inside a box furnace with heating rate of 500°C/sec to produce the final photoanode thin film with *α*-Fe_2_O_3_ phase for the water splitting process, where the rapid heating rate was accomplished by plunging the samples into the hot zone of the furnace maintained at the setting temperatures of 420~550°C. Repetition of this process yielded an H-TiNT particle thin film coated on the FTO or Fe_2_O_3_ film with approximately 700~1000 nm thickness as previously reported in our researches [30]. After the heat treatment at various conditions, the surface microstructure of the Fe_2_O_3_ thin films was observed with scanning electron microscope (SEM; S-4700, Hitachi) and their crystallinity was analyzed using X-ray diffractometer (XRD; D/MAX 2500, Rigaku), Raman spectroscopy (Renishow, inVia Raman microscope), UV-Vis spectroscopy (S-3100, Sinco). To measure the *I*-*V* and *C*-*V* electrochemical properties using *μ*Autolab type III potentiostat (Metrohm Autolab), a calomel electrode and a Pt wire were used as the reference and counter electrodes, respectively, when the as-prepared, heat-treated coated Fe_2_O_3_/H-TiNT composite films with various arrangements were used as the working electrode in an aqueous 1.0 M NaOH deaerated solution under irradiation of 100 mW/cm^2^ UV-Vis spectrum (Hg-Xe 200 W lamp, Super-cure, SAN-EI Electric). The measured potentials versus calomel were converted to the reversible hydrogen electrode (RHE) scale in all *I*-*V* graphs.

## 3. Results and Discussions

**Figure 1:**
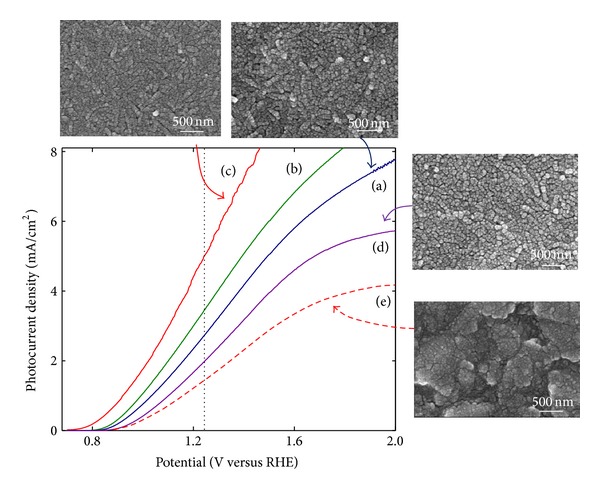
Photoelectrochemical *I-V* characteristics of Fe_2_O_3_ precursor/H-TiNT/FTO heat treated at (a) 420°C, (b) 460°C, (c) 500°C, and (d) 550°C in the air, compared to (e) Fe_2_O_3_ precursor/FTO heat treated at 500°C.

Figure 1 shows *I*-*V* photoelectrochemical data and surface morphology of the Fe_2_O_3_ precursor/(H-TiNT)/FTO samples, which had been heat treated at the predetermined temperatures of 420~550°C for 10 min. The amount of Fe_2_O_3_ in the samples was 65.48 wt% for the Fe_2_O_3_/H-TiNT/FTO and about 30 wt% for the Fe_2_O_3_/FTO, which was determined based on the *I*-*V* photoelectrochemical performance as reported in our previous study [29]. All the samples were measured in the 1.0 M NaOH solution under 100 mW/cm^2^ of UV-Vis light illumination, and the linear sweep voltammetry was in the range of 0.0~+2.0 (*V* versus RHE). The photocurrent densities were obtained by eliminating the “dark” fraction from “illumination” data, where dark data was measured in the dark room without UV light illumination. For the comparison, sample (e) without TiO_2_ interlayer was adopted from our previous work [26].

Regardless of the heat treatment temperatures, the performance improvement was observed in the samples with TiO_2_ interlayer incorporated in between Fe_2_O_3_ and FTO. In particular, sample (c) prepared under the same condition as sample (e) other than the presence of TiO_2_ interlayer film showed about 3 times increase of photocurrent density at 1.23 (*V* versus RHE) and the reduction of the onset voltage to about 0.75 V. These results suggest that the TiO_2_ interlayer can play a significant role in the efficient collection and conversion of photoenergy. The extent of performance improvement was found to be affected by the heat treatment temperature; it showed a gradual improvement with the heat treatment temperature of up to 500°C, above which it rather deteriorated. A similar result was observed with the Fe_2_O_3_/FTO samples without TiO_2_ interlayer film in our previous work [26].

Morphology of the Fe_2_O_3_/FTO sample after heat treatment at 500°C for 10 min was shown in Figure 1(e). The Fe_2_O_3_ particles were observed to form a film conformal to the FTO substrate, indicating a very thin and uniform film as noted by Oh et al. [31]. Microstructure changes of the Fe_2_O_3_ precursor/H-TiNT/FTO samples were also monitored as a function of heat treatment temperature of 420~550°C. The as-coated porous and rough H-TiNT particles with fibrous morphology as reported in our previous work [27] were broken into spherical particles through the heat treatments. It is noteworthy that the Fe_2_O_3_ particles in the Fe_2_O_3_/H-TiNT/FTO samples were relatively smaller than those in the Fe_2_O_3_/FTO sample, suggesting that the growth of the Fe_2_O_3_ particles was restrained by H-TiNT during the heat treatments. However, no noticeable microstructural differences were observed among the Fe_2_O_3_/H-TiNT/FTO samples which could explain the performance variation occurred in the samples.

**Figure 2:**
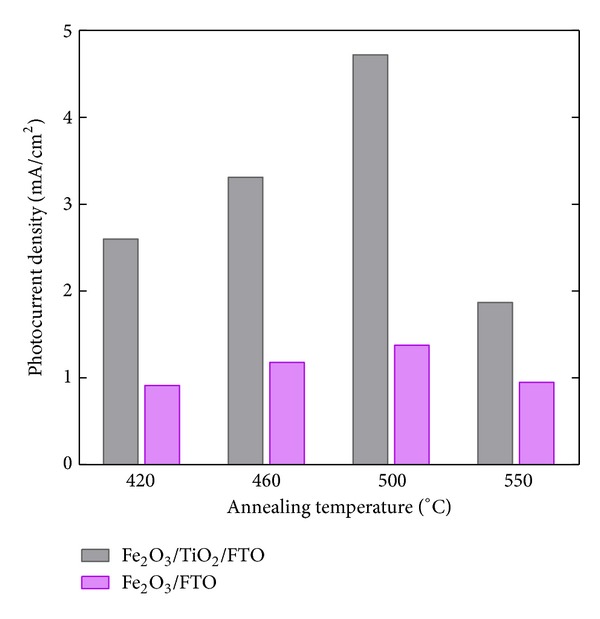
Comparison of photocurrent densities at 1.23 V versus RHE for Fe_2_O_3_/TiO_2_/FTO and Fe_2_O_3_/FTO samples with annealing temperatures.

The contribution of the TiO_2_ interlayer placed in between Fe_2_O_3_ and FTO on the photocurrent density improvement at 1.23 (*V* versus RHE) as a function of heat treatment temperature was quantitatively expressed in Figure 2. The data for the Fe_2_O_3_/FTO samples were taken as a reference from our previous work [26]. The effect of the TiO_2_ interlayer on the performance improvement was substantially increased with the temperature to the highest at 500°C, above which it rather declined.

Phase changes of the constituent materials in the samples with the heat treatments were observed in our previous work [30]. It was observed that Fe_2_O_3_ precursor was gradually transformed into *α*-Fe_2_O_3_ phase with the increase of heat treatment temperature from 420 to 550°C. However, peaks corresponding to *α*-Fe_2_O_3_ phase became weaker above 500°C. On the other hand, H-TiNT was transformed gradually but not fully into anatase-TiO_2_ phase due to the short heat treatment time of 10 min. Therefore, from the phase and photocurrent density changes of the samples, the performance improvement is considered to be closely associated with the phases present in the samples: the best performance could be obtained when H-TiNT and anatase-TiO_2_ phases coexisted with the well-developed *α*-Fe_2_O_3_ phase in the sample.

**Figure 3:**
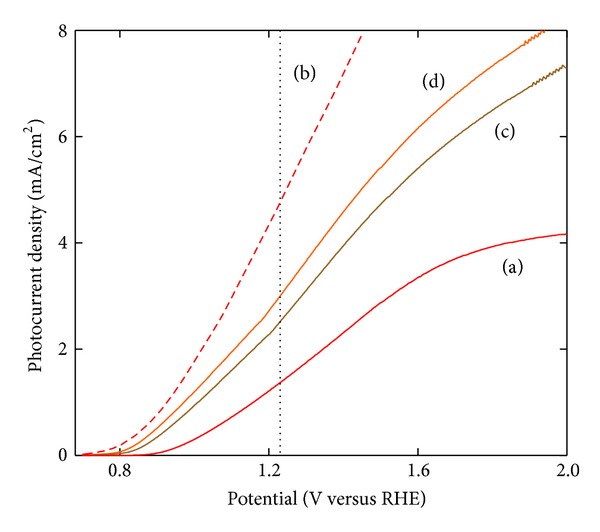
Photoelectrochemical *I-V* characteristics of the samples with the stacking structures of (a) Fe_2_O_3_/FTO, (b) Fe_2_O_3_/TiO_2_/FTO, and (c) TiO_2_/Fe_2_O_3_/FTO, which were all heat treated at 500°C for 10 min in the air. Curve (d) was obtained from Fe_2_O_3_/TiO_2_/FTO double heat treated under the same condition as above: 1st after H-TiNT coating on FTO and 2nd after Fe_2_O_3_ coating on the heat-treated H-TiNT/FTO.

Effect of the coating layers arrangement in the Fe_2_O_3_-TiO_2_-FTO samples was investigated in terms of the performance in Figure 3, in which the photocurrent densities were obtained by eliminating the “dark” fraction from “illumination” data. All the samples except sample (d) were heat treated once at 500°C for 10 min in the air following synthesis of the multilayered electrodes. Sample (d) was heat treated twice under the same condition mentioned above: once after TiNT coating on the FTO, then repeated after Fe_2_O_3_ coating on the heat-treated TiO_2_/FTO layer. Regardless of the location of TiO_2_ layer, above or below Fe_2_O_3_ layer (Fe_2_O_3_/TiO_2_/FTO (Figures 3(b) and 3(d)) or TiO_2_/Fe_2_O_3_/FTO (Figure 3(c))), samples containing TiO_2_ layer (Figures 3(b), 3(c), and 3(d)) showed much better performance compared to that (Figure 3(a)) without TiO_2_ layer, increased photocurrent density as well as reduced onset voltage.

**Figure 4:**
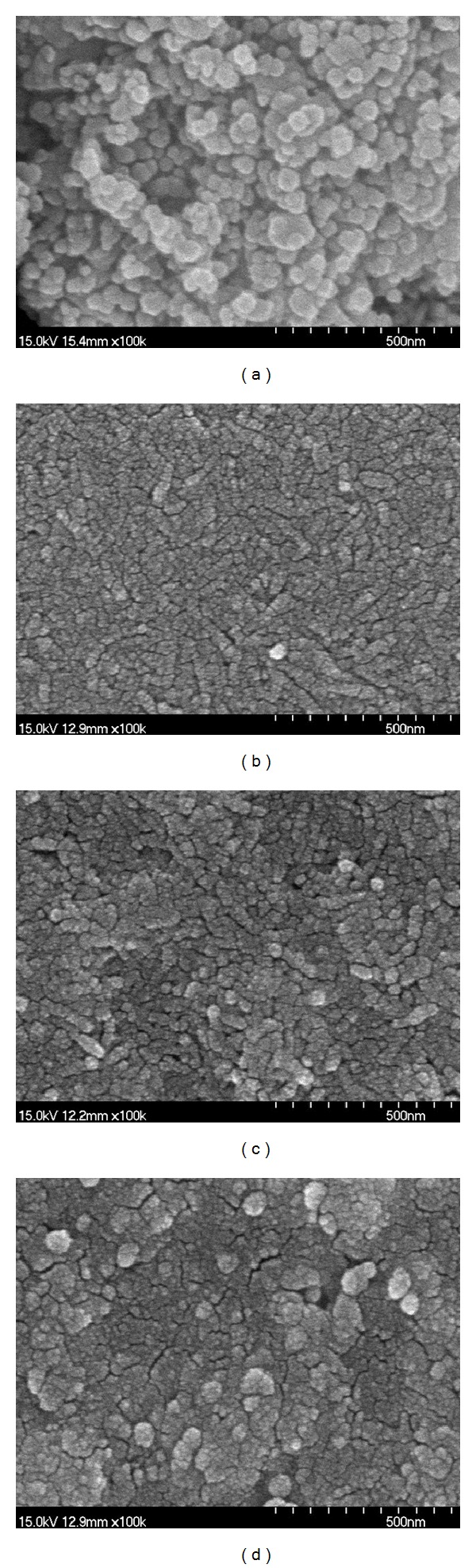
SEM photos of (a) Fe(NO_3_)_3_ powders heat treated at 500°C for 10 min, and (b), (c), (d) correspond to Figures 3(b), 3(c), and 3(d), respectively.

Microstructure observed in Figure 4 suggested that film uniformity along with the controlled particles size could play an important role for the performance improvement, Fe_2_O_3_/TiO_2_/FTO sample (Figure 4(b)) with the best performance consisted of smaller particles with high uniformity than sample (c) of TiO_2_/Fe_2_O_3_/FTO. Double heat-treated sample (d) of Fe_2_O_3_/TiO_2_/FTO showed an inferior performance to the corresponding sample (b) with the same layer structure, which was annealed only one time. This result also confirmed the importance of microstructure to the performance; the poor microstructure with agglomerated particles and cracked surface after the double heat treatment as shown in sample (d) adversely affected the performance of the sample. On the other hand, Figure 4(a) shows the Fe_2_O_3_ precursor powders becoming much larger when heat treated at 500°C for 10 min, compared to the Fe_2_O_3_ particles existing together with the TiO_2_ in the case of Figures 4(b)–4(d). These observations are consistent with the results of Figure 1, which showed the restrained growth of the Fe_2_O_3_ particles by H-TiNT during the heat treatment.

**Figure 5:**
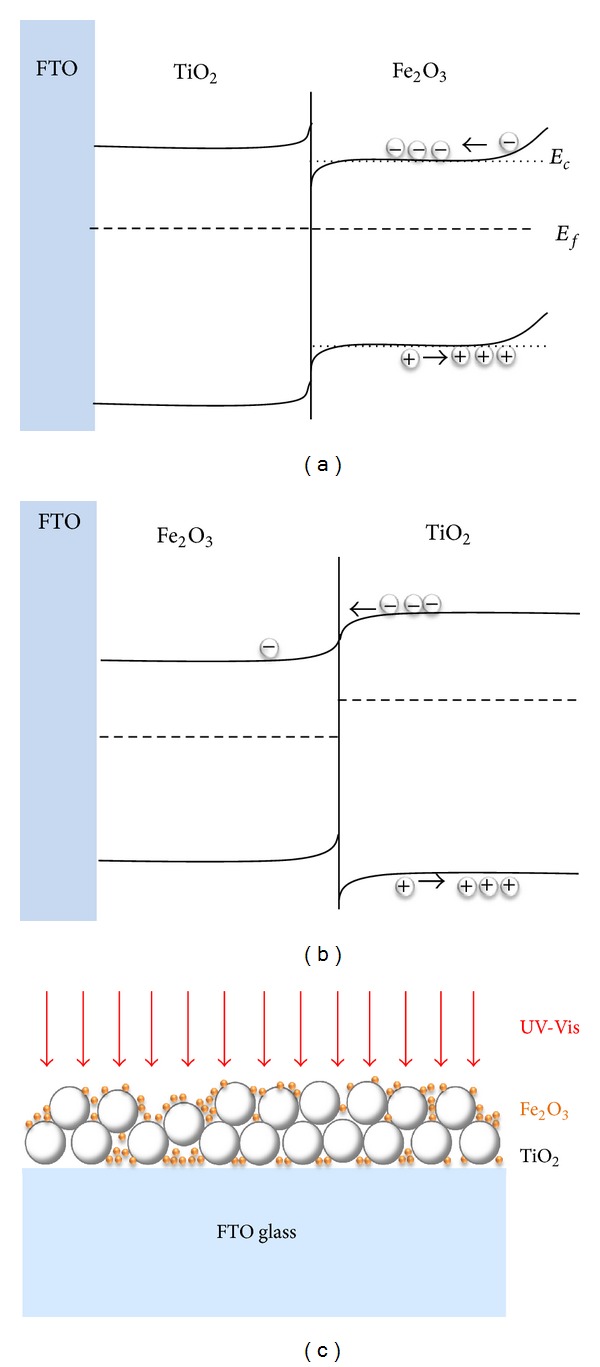
Energy band diagrams of (a) Fe_2_O_3_/TiO_2_/FTO and (b) TiO_2_/Fe_2_O_3_/FTO photoanode and (c) schematic microstructure of Fe_2_O_3_-TiO_2_-FTO.

It is noteworthy that among the samples with TiO_2_ layer, the sample (Figures 4(b) and 4(d)) with the TiO_2_ layer in between Fe_2_O_3_ and FTO layer showed better result than the sample (Figure 4(c)) having the TiO_2_ layer above Fe_2_O_3_ layer. These results were discussed in terms of energy band structure and microstructure. Energy band diagrams of the Fe_2_O_3_/TiO_2_/FTO and TiO_2_/Fe_2_O_3_/FTO samples without UV-Vis light irradiation were schematically drawn in Figures 5(a) and 5(b), respectively. It was proposed by Wang et al. that a photoelectrode with TiO_2_ based film such as SrTiO_3_ located above Fe_2_O_3_ film was a favorable structure for electrons transfer from the energy band diagram consideration [32]. Their claim seems to be reasonable from the comparison of the energy band diagrams when being not under UV-Vis light. However, our results showed that the electrons generated on the Fe_2_O_3_ layer in the Fe_2_O_3_/TiO_2_/FTO photoanode could be transferred to the TiO_2_/FTO when being under the UV-Vis light irradiation by overcoming the discontinuity of the conduction bands.

On the other hand, the microstructure of the Fe_2_O_3_/TiO_2_/Fe_2_O_3_ sample synthesized for the current work was also carefully considered. While synthesizing the Fe_2_O_3_/TiO_2_/FTO sample, some of the Fe_2_O_3_ nanoparticles could be infiltrated to the bottom FTO substrate through TiO_2_ particles when TiNT/FTO was placed in the precursor solution of Fe_2_O_3_. As a result, Fe_2_O_3_ nanoparticles could also be present in the middle TiO_2_ and the bottom FTO layer as depicted in Figure 5(c). Thus, our sample of Fe_2_O_3_/TiO_2_/FTO seemed actually to have an energy band diagram combining both of Figures 5(a) and 5(b), indicating that the photoanode with Fe_2_O_3_ nanoparticles present even in the middle and bottom substrate is preferable for the performance enhancement.

**Figure 6:**
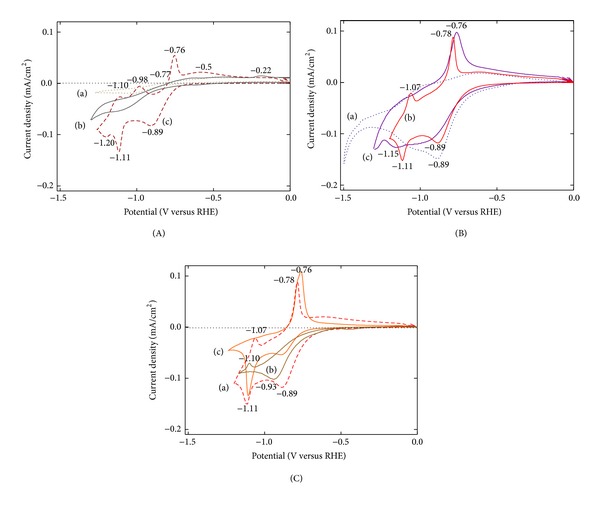
CV characteristics measured under 100 mW/cm^2^ UV-Vis illumination: (A) (a) FTO glass, (b) TiO_2_/FTO, and (c) Fe_2_O_3_/FTO samples were investigated after heat treatment at 500°C for 10 min in the air, (B) Fe_2_O_3_/TiO_2_/FTO samples were become heat treated for 10 min in the air at (a) 420°C, (b) 500°C, and (c) 550°C, (C) (a) Fe_2_O_3_/TiO_2_/FTO and (b) TiO_2_/Fe_2_O_3_/FTO heat treated at 500°C for 10 min in the air, and (c) Fe_2_O_3_/TiO_2_/FTO sample double heat treated, corresponding to (d) in Figure 3.

Oxidation-reduction reactions for the selected photoanode samples were observed by using cyclic voltammetry (CV) to investigate the effect of the coating sequence of constituent films and heat treatment condition on the photoelectrode performance. CV data for the samples of FTO glass, TiO_2_/FTO, and Fe_2_O_3_/FTO were obtained as a reference in Figures 6(A)-(a), 6(A)-(b), and 6(A)-(c), respectively. As expected the sample including Fe2O3 showed active reactions with the applied potential. According to the data (Figure 6(B)) from the Fe_2_O_3_/TiO_2_/FTO samples heat treated at the various temperature of 420 ~ 550°C for 10 min, the sample heat treated at 500°C showed multiple oxidation-reduction peaks, contributing to higher photocurrent density. These results were found to be consistent with *I*-*V* data of the samples described in Figure 1 where the sample heat-treated at 500°C showed best performance. The sample of Fe_2_O_3_/TiO_2_/FTO which showed best result after heat treatment at 500°C was then compared with TiO_2_/Fe_2_O_3_/FTO sample to see the effect of the location of TiO_2_ layer placed in the photoanode, which was also heat treated under the same condition. These samples showed a clear contrast in the results as shown in Figures 6(C)-(a) and 6(C)-(b), respectively: Fe_2_O_3_/TiO_2_/FTO sample produced more and clear oxidation-reduction peaks. On the other hand, the sample of Fe_2_O_3_/TiO_2_/FTO, which was heat treated twice after each coating of TiO_2_ and Fe_2_O_3_ layers, showed an intermediate performance (Figure 6(C)-(c)). These results were all well consistent with the *I*-*V* data in Figure 3 where the sample of Fe_2_O_3_/TiO_2_/FTO heat treated once (Figure 3(b)) at 500°C showed best performance followed by the sample double heat treated (Figure 3(d)) and TiO_2_/Fe_2_O_3_/FTO sample (Figure 3(c)).

## 4. Conclusions

Fe_2_O_3_-TiO_2_ based photoanodes for water splitting were synthesized on the FTO substrate and their performance results were understood from the microstructure and energy band aspects. Comparatively, the photoanode (Fe_2_O_3_/TiO_2_/FTO) comprising top layer of *α*-Fe_2_O_3_ nanoparticles along with the interlayer having mixed phases of H-TiNT/anatase-TiO_2_ showed best performance. The nanoscaled Fe_2_O_3_ particles with high uniformity were observed to contribute to the performance enhancement. In addition, the presence of the Fe_2_O_3_ nanoparticles in the middle and bottom layers caused by the infiltration of the precursor solution of Fe_2_O_3_ during synthesis seemed to modify the energy band structure to more favorable one for efficient electrons transfer. Our current results suggest that the application of the TiO_2_ interlayer, together with optimized amount of *α*-Fe_2_O_3_ nanoparticles present in the constituent layers, could significantly contribute to the performance improvement of the conventional Fe_2_O_3_ photoanode.
